# Investigating Users’ Attitudes Toward Automated Smartwatch Cardiac Arrest Detection: Cross-Sectional Survey Study

**DOI:** 10.2196/57574

**Published:** 2024-07-25

**Authors:** Wisse M F van den Beuken, Hans van Schuppen, Derya Demirtas, Vokko P van Halm, Patrick van der Geest, Stephan A Loer, Lothar A Schwarte, Patrick Schober

**Affiliations:** 1 Department of Anesthesiology, Amsterdam UMC, Amsterdam, Netherlands; 2 Helicopter Emergency Medical Service Lifeliner 1, Amsterdam, Netherlands; 3 Center for Healthcare Operations Improvement and Research, University of Twente, Enschede, Netherlands; 4 Industrial Engineering and Business Information Systems, University of Twente, Enschede, Netherlands; 5 Department of Cardiology, Amsterdam UMC, Amsterdam, Netherlands; 6 Ambulance Rotterdam-Rijnmond, Barendrecht, Netherlands; 7 Department of Intensive Care Medicine, Spaarne Gasthuis, Haarlem, Netherlands

**Keywords:** out-of-hospital cardiac arrest, wearables, wearable, digital health, smartwatch, automated cardiac arrest detection, emergency medicine, emergency, cardiology, heart, cardiac, cross sectional, survey, surveys, questionnaire, questionnaires, experience, experiences, attitude, attitudes, opinion, perception, perceptions, perspective, perspectives, acceptance, adoption, willingness, intent, intention

## Abstract

**Background:**

Out-of-hospital cardiac arrest (OHCA) is a leading cause of mortality in the developed world. Timely detection of cardiac arrest and prompt activation of emergency medical services (EMS) are essential, yet challenging. Automated cardiac arrest detection using sensor signals from smartwatches has the potential to shorten the interval between cardiac arrest and activation of EMS, thereby increasing the likelihood of survival.

**Objective:**

This cross-sectional survey study aims to investigate users’ perspectives on aspects of continuous monitoring such as privacy and data protection, as well as other implications, and to collect insights into their attitudes toward the technology.

**Methods:**

We conducted a cross-sectional web-based survey in the Netherlands among 2 groups of potential users of automated cardiac arrest technology: consumers who already own a smartwatch and patients at risk of cardiac arrest. Surveys primarily consisted of closed-ended questions with some additional open-ended questions to provide supplementary insight. The quantitative data were analyzed descriptively, and a content analysis of the open-ended questions was conducted.

**Results:**

In the consumer group (n=1005), 90.2% (n=906; 95% CI 88.1%-91.9%) of participants expressed an interest in the technology, and 89% (n=1196; 95% CI 87.3%-90.7%) of the patient group (n=1344) showed interest. More than 75% (consumer group: n= 756; patient group: n=1004) of the participants in both groups indicated they were willing to use the technology. The main concerns raised by participants regarding the technology included privacy, data protection, reliability, and accessibility.

**Conclusions:**

The vast majority of potential users expressed a strong interest in and positive attitude toward automated cardiac arrest detection using smartwatch technology. However, a number of concerns were identified, which should be addressed in the development and implementation process to optimize acceptance and effectiveness of the technology.

## Introduction

Out-of-hospital cardiac arrest (OHCA) is a leading cause of mortality in the developed world [1,2]. The chain of survival after OHCA starts with timely detection of the cardiac arrest and prompt activation of emergency medical services (EMS) [3,4]. While this step is crucial to prevent the death of the patient, it is also the most fragile link in the chain of survival because it requires the presence of a witness. Reliance on bystander activation often introduces a significant delay that markedly reduces the chances of survival [5-7].

Several research groups, including our own, are developing a possible technical solution to automatically detect OHCA and to automate the activation of EMS using wearables and smart devices [8-10]. Smartwatches are computing devices that resemble wristwatches, with functionalities comparable to smartphones. Smartwatches are one of the most prevalent wearable technologies [11] and incorporate a wide array of sensors. Among these sensors are GPS and photoplethysmography (PPG). GPS is a global navigation satellite system that provides location, velocity, and time. This could potentially be used to track the location of patients experiencing cardiac arrest. PPG is used to detect changes in light absorption due to pulsatile blood flow [12] and hence can be used to measure the heartbeat, allowing smartwatches to accurately detect cardiac arrhythmias [13-15]. PPG and other sensors integrated in smartwatches could also potentially be used to detect cardiac arrest by measuring the cessation of pulsatile blood flow.

Such a technical solution, which is currently being developed, should be well aligned with the needs of its potential users in order to enable successful implementation [8]. We therefore aim to investigate users’ perspectives on aspects of continuous monitoring such as privacy and data protection, as well as other implications, and to collect insights into their attitude toward the technology.

## Methods

### Study Design

We conducted a cross-sectional web-based survey aiming to investigate the perceptions and attitudes of potential users toward automated cardiac arrest diagnosis using smartwatches. We identified 2 groups of potential users: the first was consumers who already own a smartwatch (the consumer group), as these individuals could instantly make use of this technology as soon as it becomes available. The second was patients at increased risk of experiencing cardiac arrest (the patient group), as these individuals can potentially benefit the most from using this technology. A survey was deployed among both groups as described below, and participants had up to 4 weeks to respond. Data were collected between October 27, 2022, and March 17, 2023.

### Ethical Considerations

The study was assessed by the Medical Ethics Review Committee of VU University Medical Center (2022.0544), which declared on November 29, 2022, that the study was not subject to the Medical Research Involving Human Subjects Act (WMO), such that formal approval was not required. Participants received written information about the purpose of the research and consented to the use of their provided answers for research purposes. Participants in the consumer group were compensated with €0.30 to €0.50 (US $0.32 to $0.53) for their participation. Participants in the patient group did not receive any compensation. The data collected from both groups were completely anonymous.

### Participant Recruitment

#### Consumer Group

The consumers were recruited by a leading Dutch market research agency, Markteffect. This agency has several panels comprising approximately 225,000 consumers from different sectors in the Netherlands. For our survey, we recruited consumers who were aged 18 years or older and owned a smartwatch.

#### Patient Group

The at-risk patients were recruited through the health panel of the Netherlands Patient Federation (NPF). This health panel comprises approximately 23,000 individuals from different patient associations. All patients had to be aged at least 18 years and at increased risk for cardiac arrest based on self-reported medical history and comorbidities. Patients were considered at increased risk of cardiac arrest if they had 1 or more of the following: cardiovascular disease (eg, hypertension, heart failure, angina pectoris, myocardial infarction), severe renal insufficiencies, diabetes mellitus, cerebrovascular accident, or severe pulmonary disease (eg, chronic obstructive pulmonary disease or lung emphysema).

### Sample Size Considerations

An a priori sample size analysis revealed that a minimum sample size of 385 participants would be needed to attain a margin of error of no more than 5% at a 95% confidence level. To attain an even higher precision while still allowing for dropouts and subgroup analyses, we targeted approximately 1000 participants per user group.

### Survey Development and Data Collection

The surveys were developed by WMFvdB and PS in collaboration with the research experts from Markteffect and the NPF. Both surveys underwent a comprehensive review and testing by members of our research group and Markteffect or the NPF, respectively, and the surveys can be found in Multimedia Appendix 1. Both surveys largely comprised the same introductory text and questions, but slight modifications were made to tailor the surveys to each group. The surveys included an introductory text explaining the prevalence of witnessed and unwitnessed OHCAs in the Netherlands, emphasizing the need for an alerting system to ensure prompt intervention in case of cardiac arrest. We explained that we are currently developing a novel technology that could potentially address this issue, as it is capable of automatically diagnosing cardiac arrest using smartwatches. We underscored our interest in obtaining their opinions and perspectives on this technology. If participants agreed to participate by continuing to the online survey, we would proceed to ask questions regarding automated cardiac arrest diagnosis using smartwatches. In addition, we asked questions about gender, age, income, and other demographic characteristics. The questions primarily consisted of closed-ended questions and included 5-point Likert-scale questions to assess their agreement with specific aspects of the technology. Limited open-ended questions were provided to enable supplementary insights. Markteffect hosted and distributed the online survey to the consumer group using Collecthor (Collecthor BV). The online survey sent to the patient group was created and hosted Castor Electronic Data Capture (Ciwit BV). The NPF distributed the survey to the patients who were at increased risk of cardiac arrest and had indicated interest to participate in the survey.

### Data Screening and Statistical Analysis

Markteffect cleaned the consumer data set, removing surveys with any of the following characteristics, according to their internal standard operating procedures: (1) speeders—surveys that were completed within an exceptionally implausibly fast timeframe; (2) double respondents—multiple surveys submitted by the same individual; (3) straight liners—participants who consistently selected the same answer choice (eg, always chose a neutral response or always chose the first option) or did not answer the questions; and (4) surveys with consistent unintelligible language. The cleaned data set was provided for further analysis. The data from at-risk patients were cleaned by removing completely empty surveys, speeders, and surveys in which less than 50% of the questions were completed.

Quantitative data were analyzed descriptively. Measures of central tendency, measures of variation, and measures of distribution were calculated [16]. The analyses were performed using R (version 4.2.1) and RStudio (version 2022.2.3.492; R Foundation for Statistical Computing).

A content analysis of the open-ended questions was conducted to provide deeper insights into the participants’ perspectives. WMFvdB created a coding framework consisting of inductive and deductive codes. The coding was reviewed by PS and any disagreements were resolved in consensus. The codes were categorized to identify emerging themes. The content analysis was performed using MAXQDA 2022 (VERBI Software).

## Results

### Quantitative Analyses

**Figure 1.**
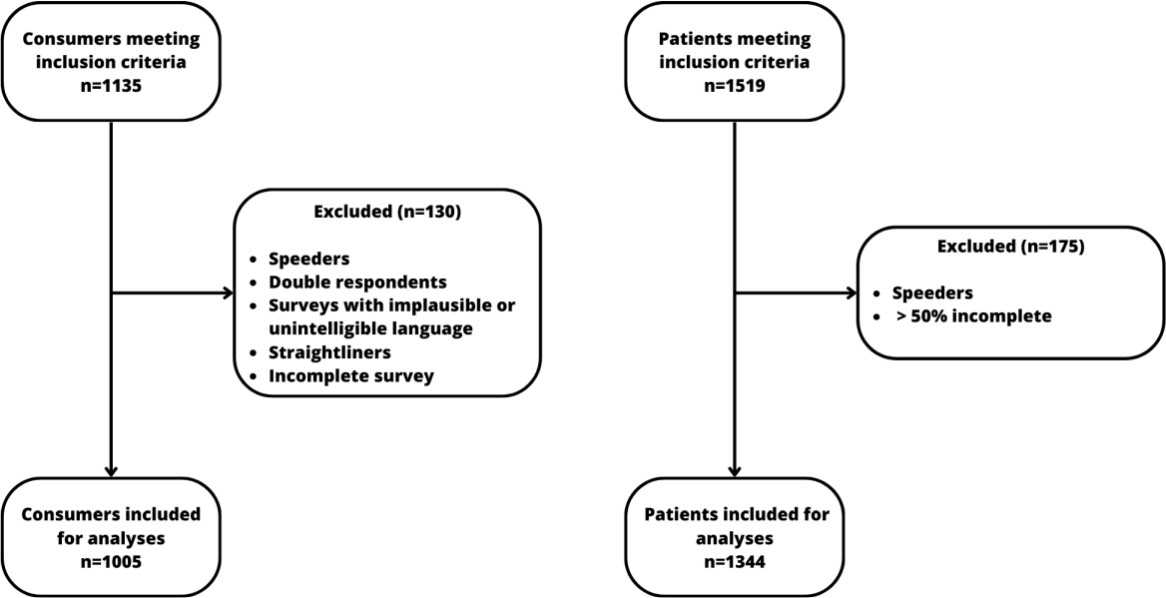
Flowchart of participant inclusion.

In the consumer group and the patient group, 1135 and 1519 participants, respectively, met the inclusion criteria. In both groups, 88.5% (n=1005 and n=1344, respectively) of the participants were included for analysis (Figure 1).

**Figure 2.**
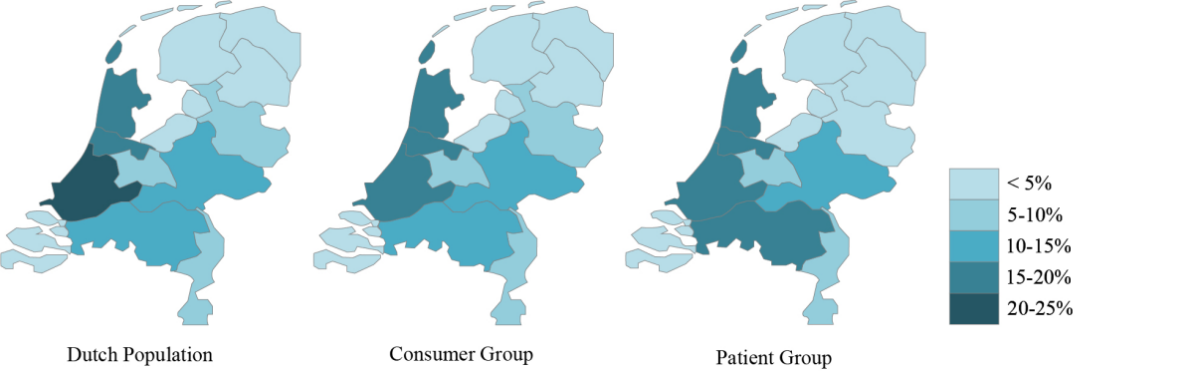
Population density in percentages. Data for the Dutch population for 2021 comes from the Central Bureau of Statistics [18].

The mean age in the consumer group was 48.6 (SD 13.2) years, and 48.7% (n=489) of the population was male. In the patient group, the mean age was 67.7 (SD 10.0) years, and 56.4% (n=750) of the population was male. Regarding level of education, smartwatch preference, and ethnic background, both groups followed a similar pattern. In the consumer and patient groups, 58.5% (n=588) and 56.1% (n=731), respectively, had a higher level of education. Apple and Samsung smartwatches were most prevalent, and the majority of the population had a Dutch background (Table 1). Both groups had a similar geographical distribution throughout the Netherlands as the general Dutch population (Figure 2).

**Table 1.** Population characteristics.

		Consumers (n=1005)	Patients (n=1344)	Dutch population^a^ (n=17,475,415)
Age (years), mean (SD)		48.6 (13.2)	67.7 (10.0)	42.3 (N/A^b^)
Male sex, n (%)		489 (48.7)	750 (56.4)	8,686,536 (49.7)
Higher level of education, n (%)		588 (58.5)	731 (56.1)	6,203,772 (35.5)
**Smartwatch brands n (%)**				
	Apple	258 (25.7)	112 (25.5)	N/A
	Samsung	252 (25.1)	107(24.4)	N/A
	Garmin	144 (14.3)	48 (10.9)	N/A
	Fitbit	127 (12.6)	63 (14.4)	N/A
	Other	224 (22.3)	120 (27.3)	N/A
**Household income** ^c^ **, n (%)**				
	<€1600 per month	50 (5)	93 (7.5)	N/A
	€1600-€2600 per month	97 (9.7)	204 (16.5)	N/A
	€2600-€3000 per per month	119 (11.8)	211 (17.1)	N/A
	€3000-€4000 per month	228 (22.7)	229 (18.5)	N/A
	€4000-€8000 per month	233 (23.2)	227 (18.4)	N/A
	>€8000 per month	64 (6.4)	45 (3.6)	N/A
	I do not know/I prefer not to state	214 (21.2)	226 (18.4)	N/A
**Region of origin n (%)**				
	Dutch	947 (94.2)	1164 (96.1)	N/A
	Western	23 (2.3)	24 (2)	N/A
	Non-Western	24 (2.4)	10 (0.8)	N/A
	I prefer not to state	11 (1.1)	10 (0.8)	N/A
**Medical history, n (%)**				
	High blood pressure	N/A	864 (64.3)	N/A
	Diabetes mellitus	N/A	324 (24.1)	N/A
	Cardiovascular disease	N/A	752 (56)	N/A
	Severe renal disease	N/A	59 (4.4)	N/A
	Cerebrovascular accident	N/A	109 (8.1)	N/A
	Severe lung disease	N/A	274 (20.4)	N/A
	History of cardiac arrest	N/A	78 (6.2)	N/A
	Other	N/A	204 (15.2)	N/A

a Data from 2021, acquired from the Central Bureau of Statistics (CBS) in the Netherlands [17,18].

b N/A: not applicable.

c In 2021, according to CBS, the median primary income of a household in the Netherlands was €3525 per month [19]. An exchange rate of €1=US $1.06054 applied at the time of the study.

**Figure 3.**
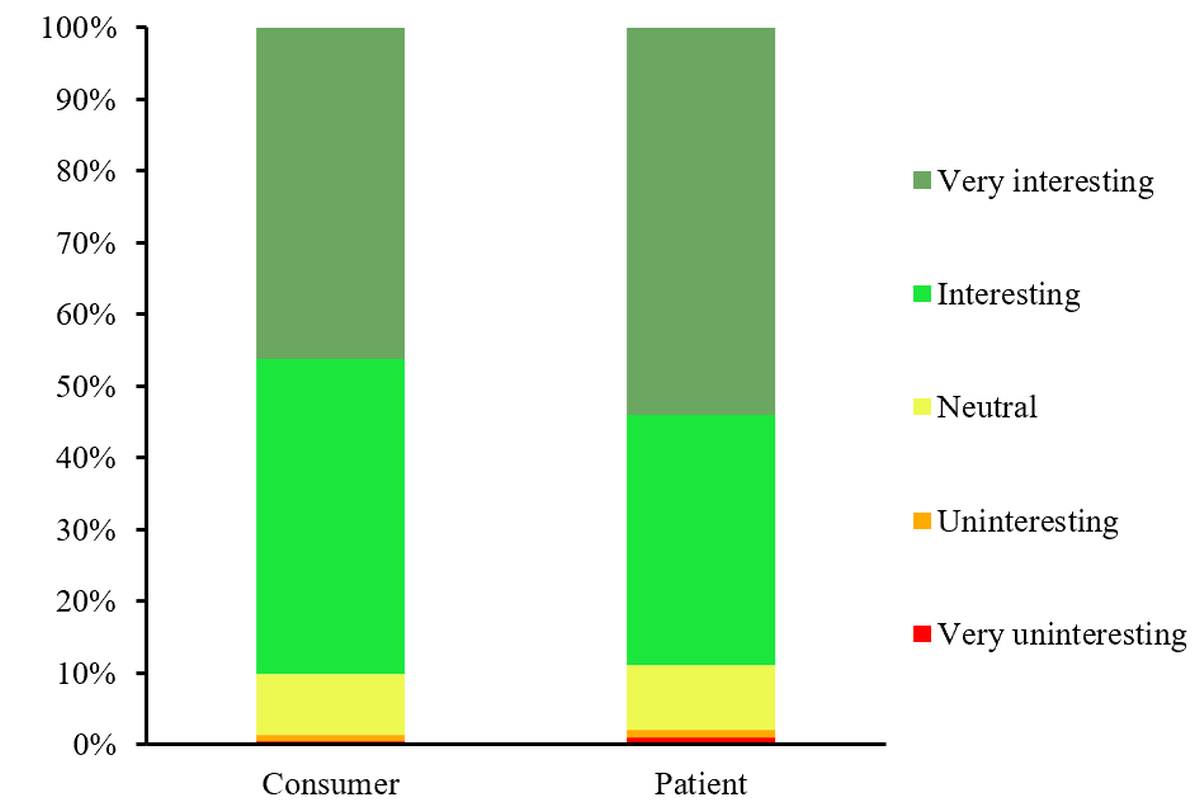
Participants’ interest in the technology according to a 5-point Likert scale.

**Figure 4.**
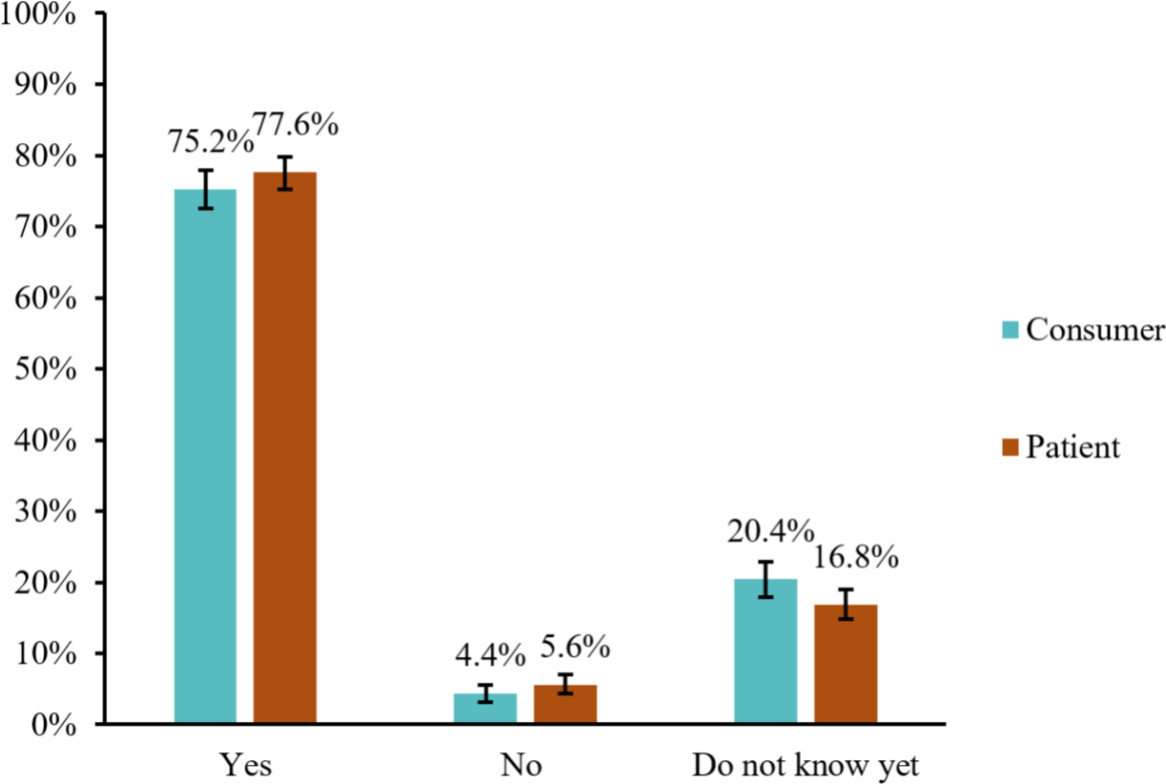
Participants’ willingness to use the technology. Error bars represent 95% CIs. Consumer group n=1005, patient group n=1294.

**Figure 5.**
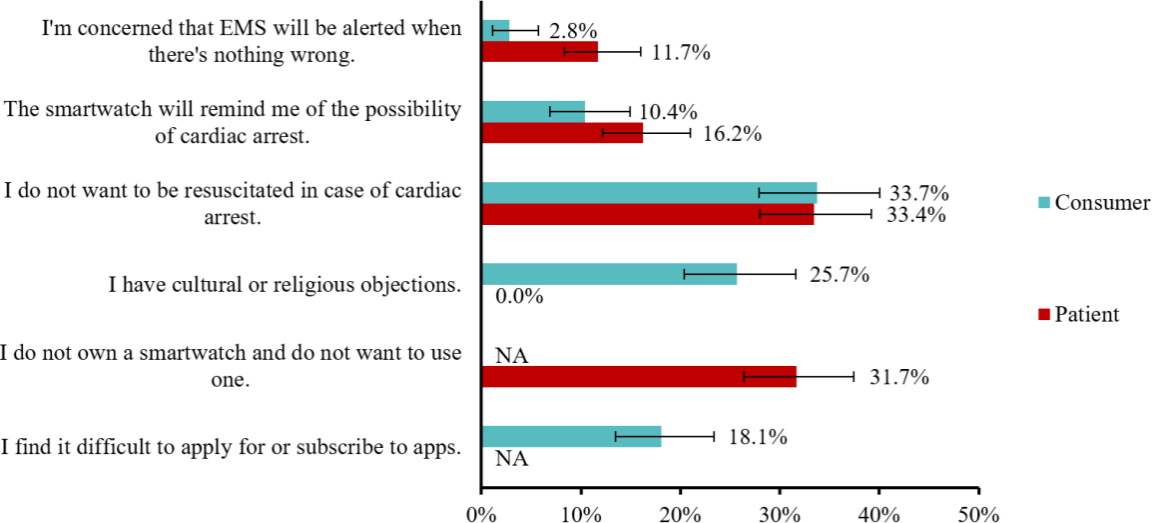
Most cited reasons for abstaining from or expressing uncertainty about adopting the technology. Error bars represent 95% CIs. Consumer group n=249, patient group n=290. EMS: emergency medical services.

Both groups expressed interest in the technology; 90.2% (n=906; 95% CI 88.1%-91.9%) of the consumer group and 89% (n=1196; 95% CI 87.3%-90.7%) of the patient group considered the technology as “interesting” or “very interesting” (Figure 3). Moreover, 75.2% (n=756; 95% CI 72.5%-77.9%) of the participants in the consumer group and 77.6% (n=1004; 95% CI 75.3%-79.8%) of the patient group indicated their willingness to use the technology (Figure 4). The most frequently cited reason for abstaining from or expressing uncertainty about adopting the technology in both groups was “not wanting to be resuscitated.” The second most common reason given by the consumers was cultural or religious objections, whereas none of the patients provided this as a reason for expressing uncertainty or abstaining from using the technology. In the patient group, 31.7% (n=92) of participants who had indicated abstaining from or expressed uncertainty about adopting the technology stated not wanting to use a smartwatch as a reason (Figure 5).

**Figure 6.**
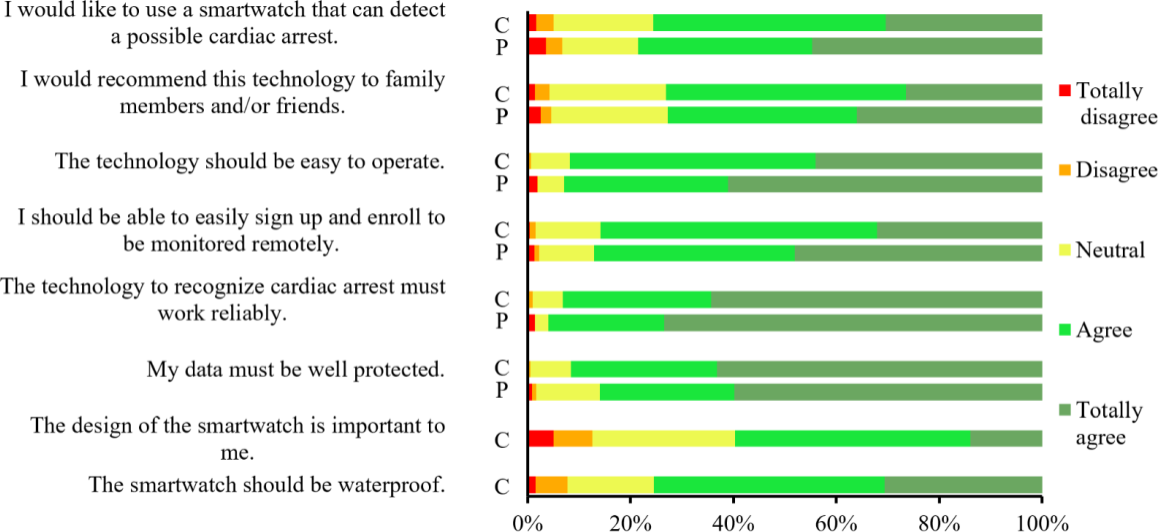
Participants’ agreement with general statements regarding the technology, ranked according to a 5-point Likert scale. C: consumer group; P: patient group.

In both groups, around 75% (consumer group: n=760; patient group: n=1045) agreed that they would like to use a smartwatch that can be used to detect cardiac arrests, about 72% (consumer group: n=735; patient group: n=964) agreed they would recommend the technology to family and friends, and around 86% (consumer group: n=863; patient group: n=1116) agreed that it should be easy to sign up to use the technology. Both groups agreed (>93%) that the technology should be reliable (consumer group: n=936; patient group: n=1204), and agreed (around 92%) that the technology should be easy to operate (consumer group: n= 923; patient group: n=1223). The consumer group felt a little more strongly that data should be well protected; 91.6% (n=920) agreed compared to 86% (n=1071) in the patient group (Figure 6).

Participants were also surveyed regarding their willingness to make monthly payments for the technology, expressed in euros. The median amount the consumer group and patient group were willing to allocate, given they indicated that they would use the technology, was €5.0 (IQR €2.0‐€10.0) and €10.0 (IQR €5.0‐€20.0), respectively, per month (an exchange rate of €1 = US $1.06054 applied at the time of the study).

### Content Analyses

The open-ended questions were categorized into the most prevalent themes, as summarized below. Table 2 provides a quantitative overview of the content analysis.

**Table 2.** Content analysis.^a^

Themes and subthemes		Consumers, n	Patients, n	Total, n
**Life-saving potential**				
	Overall	278	402	680
	Time	107	257	364
	Experience/medical history	72	195	267
**Peace of mind**				
	Overall	49	72	121
	Reduce stress	39	59	98
	Induce stress	10	13	23
Prevention		48	92	140
**Affordability and accessibility**				
	Overall	96	145	241
	Health inequity	22	53	75
	Ease of use	30	44	74
Accuracy and reliability		55	59	114
Data and privacy protection		24	11	35

a Participants could give answers that were applicable to multiple themes and subthemes. The table shows the number of individuals that mentioned the specific theme in the open-ended questions.

#### Life-Saving Potential

Both groups acknowledged the potential life-saving capabilities of the technology; this was mentioned by 680 participants. Participants underscored the importance of swift intervention during critical events such as cardiac arrest and expressed that this technology could help shorten the time to resuscitation by EMS; 364 participants mentioned this. They acknowledged that rapid intervention is associated with increased survival and recognized the potential benefit from using automated cardiac arrest detection.

In total, 267 participants mentioned their personal experiences or medical history. Some shared accounts of friends or family members who experienced cardiac arrest, some of whom did not survive. These experiences influenced their favorable perception toward automated cardiac arrest diagnosis. Additionally, participants, especially in the patient group, often referred to their own medical history, noting their increased risk of cardiac arrest. They expressed a strong desire to use every tool available to enhance their chances of survival in such an event.

#### Peace of Mind

A total of 98 participants mentioned that the technology could also provide peace of mind for users, offering the assurance that EMS will be alerted in the event of cardiac arrest, even if no witnesses are present. Potential users mentioned an increased feeling of security and confidence if such a technology were available, potentially leading to greater physical activity. However, 23 individuals also expressed concerns that this technology could serve as a constant reminder of the possibility of cardiac arrest, potentially leading to stress.

#### Prevention

A total of 140 respondents also mentioned that such a technology could potentially be used for preventive purposes, such as detecting a cardiac problem before it manifests in cardiac arrest. Participants also indicated that using this technology may raise awareness for heart problems and may also promote a healthier lifestyle.

#### Affordability and Accessibility

A total of 241 individuals expressed concerns about, or mentioned the importance of, the accessibility and affordability of the technology. Among them, 75 highlighted that disparities in access could potentially lead to or exacerbate health care inequities. The participants indicated that researchers and product manufacturers should identify solutions for widespread accessibility.

Moreover, participants mentioned the importance of integrating the technology into standard health insurance as an option to enhance accessibility. Finally, to increase accessibility, the technology should be intuitive, simple, and easy to use. This was emphasized by 74 individuals. This is especially important when making the technology accessible for users with a low level of technical proficiency.

#### Accuracy and Reliability

A total of 114 participants expressed concerns regarding the accuracy and reliability of the technology. They emphasized that thorough testing is needed to minimize false alarms. False alarms could potentially strain the existing health care system, induce anxiety, and erode trust in the technology.

#### Data and Privacy Protection

Another concern raised by 35 participants was related to data collection and the secure handling of medical and personal data. Mainly, the consumers placed significant emphasis on safeguarding their data and privacy and the need to obtain consent regarding data collection while ensuring that only essential data are collected. Some participants expressed reservations about the involvement of smartwatch companies in managing medical data.

## Discussion

### Principal Findings

We assessed attitudes and perceptions of potential users toward automated cardiac arrest detection using smartwatch sensor data. We found that the vast majority of the participants expressed their willingness to adopt this innovative technology. However, 1 of 20 individuals indicated that they did not want to adopt this technology, and a considerable number of individuals were yet undecided. Our findings revealed several barriers and concerns that warrant careful consideration.

Previous research assessed the acceptability of the use of wrist-worn wearables, mainly focusing on activity tracking and time spent using the device as a measurement of acceptance [20]. However, to our knowledge, our research is the first to focus on the perceptions of potential users and their willingness to accept automated cardiac arrest detection. Understanding users’ perspectives is imperative for research groups developing such technology, enabling them to address concerns early in the development process. Moreover, health care professionals involved in counseling patients at risk, as well as any stakeholders involved in the implementation, distribution, or marketing of the technology, should have a clear understanding of users’ perspectives. This is paramount in order to increase acceptability [21-23] and to ensure effective implementation [24].

In this context, the insights gained from this study have several important implications. One key factor contributing to reluctance in adopting the technology is that some individuals do not want to be resuscitated in the event of a cardiac arrest. Remarkably, this reluctance was not limited to older people or those with significant comorbidities in the patient group but also extended to the relatively young population in the consumer group. While there are legitimate reasons for refusing resuscitation attempts, it is likely that this reluctance partially stems from a lack of understanding about the prognosis of cardiac arrest, particularly when detected and treated early. Fear of being severely handicapped or incapacitated may have played a role. However, in the Netherlands, approximately 90%‐95% of cardiac arrest survivors are known to survive with a favorable neurologic outcome [25,26]. Education and awareness campaigns may play a pivotal role in addressing misconceptions, ensuring that potential users are well-informed about the life-saving potential of the technology [27].

In the consumer group, cultural or religious objections to adopting the smartwatch-based cardiac arrest technology were frequently reported. Although our survey did not delve into the specific nature of these objections, the evident heterogeneity among potential users highlights the need for customized product development and implementation strategies. Acknowledging and understanding the cultural and religious dynamics that influence technology adoption decisions is critical and should be investigated in more depth in future studies.

In the patient group, a significant barrier to adopting smartwatch-based cardiac arrest detection technology was the reluctance to use a smartwatch. This may partly be attributed to a lack of digital literacy. In particular, older individuals may be less accustomed to digital technology and may perceive a high complexity of operating such devices. Emphasizing simplicity and intuitive operation in the development process is therefore crucial.

Potential users also expressed concerns regarding the potentially high cost of the technology and that financial inaccessibility may exacerbate disparities in health equity [28]. It is worth noting that low socioeconomic status is associated with a higher incidence of cardiac arrest [29], suggesting that this demographic may potentially benefit the most from this technology. Therefore, future research on automatic cardiac arrest detection should include a sufficient number of participants from lower socioeconomic backgrounds. Given that low socioeconomic status is associated with a higher incidence of cardiac arrest, it is crucial to consider cost-effectiveness and affordability early in the development phase. Proactive collaboration with health insurance providers, public social welfare systems, nonprofit health care foundations, and government organizations is essential to ensure financial accessibility.

The analysis also highlighted concerns about privacy, data protection, and data use, which are common concerns when introducing novel medical technologies [30]. Governmental and regulatory bodies have been developing guidelines for software used as a medical device to ensure privacy and data protection [31-34]. For developers and researchers involved in creating such technologies, strict adherence to these regulations plays a vital role in building user trust by ensuring the robust protection of their privacy.

Another concern identified was the reliability of automated cardiac arrest detection systems. Developers are thus challenged to achieve exceptionally high levels of sensitivity and specificity [35]. The goal is to create a system capable of accurately identifying cardiac arrest events while simultaneously minimizing false alarms, which are detrimental to both user trust and system efficacy.

### Strengths and Limitations

This study features a considerable sample size encompassing a diverse range of participants who might use this technology. The questionnaire was carefully designed using the joint expertise of a leading professional market research organization, a patient federation experienced in researching patient perceptions, and a medical research group with documented experience in survey methodology [36-38].

Our research not only focuses on patients at increased risk of cardiac arrest, but also includes consumers who already own a smartwatch, which positions them to be early adopters as soon as the technology is implemented. It is noteworthy that approximately 50% of individuals experiencing a cardiac arrest have no prior history of cardiac symptoms or events, and OHCA also frequently affects middle-aged adults [39]. This underscores the applicability of this technology across a broad demographic, including those perceived as “healthy.” By encompassing both high-risk patients and regular smartwatch users, our study captures a wide spectrum of perspectives, enhancing the relevance and applicability of our findings.

We acknowledge some limitations. First, a few participants (n=30) seemed to conflate the terms cardiac arrest and heart attack, suggesting potential misunderstandings about what the technology monitors and detects. This confusion may have influenced their responses. However, both heart attack and cardiac arrest are serious medical conditions that benefit from early detection, and the rationale for continuous monitoring should logically extend from one condition to the other. This confusion also underscores the necessity for enhanced public education to improve understanding of these distinct medical events [27].

Second, the research was conducted with patients and consumers in the Dutch population, where local culture and health care systems may shape attitudes toward technology adoption [40]. This aspect has to be considered when extrapolating our results to other regions, with different health care systems or cultures.

### Conclusion

The vast majority of potential users expressed a positive attitude toward automated cardiac arrest detection using smartwatch technology. The primary concerns raised by participants included privacy, data protection, reliability, and accessibility of the technology. Despite such concerns, the vast majority indicated that they would be willing to use the technology. This indicates a strong potential user base but also underscores the importance of addressing the identified concerns to optimize acceptance and effectiveness of the technology.

## Supplementary material

10.2196/57574 Multimedia Appendix 1 Translated surveys.

## Abbreviations

CBS: Central Bureau of Statistics
EMS: emergency medical services
NPF: Netherlands Patient Federation
OHCA: out-of-hospital cardiac arrest
PPG: photoplethysmography
