# Supplementary material for: Investigating Users’ Attitudes Toward Automated Smartwatch Cardiac Arrest Detection: Cross-Sectional Survey Study
Source: JMIR Hum Factors. 2024 Jul 25;11:e57574. doi: 10.2196/57574 (PMC11292589; doi:10.2196/57574)
Supplement: Multimedia Appendix 1 [file humanfactors-v11-e57574-s001.docx]

Supplementary **material**

# Legend

- A. Translated Survey Consumers
- B. Translated Survey Patients

# A. Translated Survey Consumers

**Part 1: Screening**

S000

What is your age?

<Open answer field> years

Screenout if under 18 years old

S001

What are the four digits of your zip code?

<Open answer field>

S002

What is your gender?

- Male

- Female

- Other than above

S003

Which of the following items do you own?

Show options randomly

- Smartwatch

- Smartphone

- Earphones

- Laptop

- Earphones

- Tablet

- None of the above -> screenout

**Part 2 Introduction**

For research on a new technology to use smartwatches to recognize cardiac arrest, we are looking for smartwatch users who would like to share their views on this. The text in the next paragraph gives a little more background information if you are interested, but can also be skipped.

In the Netherlands, 17,000 people are struck by cardiac arrest outside the hospital every year. A large number of these people die because emergency services are called too late or not at all (if there are no witnesses). With every minute delay until the right treatment is started, the chance of making a good recovery drops significantly. This is why researchers at the Amsterdam UMC are currently working on developing a new technology in which a cardiac arrest is recognized through a smartwatch and emergency services (ambulance service and lay rescuers) are automatically and immediately called in. Their vision is that in the future, anyone who owns a smartwatch and a smartphone can sign up to be monitored remotely so that a cardiac arrest is recognized immediately. The goal is for this to work with many different smartwatches and not require buying a particular model. This survey is being conducted to get a better idea of what current smartwatch users think of these plans and what specifically we should consider while developing the technology.

Completing this survey will take no more than 10 minutes of your time. Participation in the survey is completely voluntary and anonymous. By completing the survey, you declare your consent to the use of the information for research. The data will be processed confidentially. None of the questions are mandatory. If you do not want to fill in an answer to a particular question, you do not have to.

Thank you very much for your cooperation!

**Part 3: Perception**

V001a

What do you think about the idea of smartwatches being used to recognize cardiac arrest**?**

| Not interesting at all | Not interesting | Neutral | Interesting | Very interresting |
| --- | --- | --- | --- | --- |
|  |  |  |  |  |

V001b

Can you explain your answer?

<open answer field>

V002

If it is possible in the future to reliably recognize cardiac arrest through a smartwatch, would you want to take advantage of this?

- Yes

- No

- Do not know yet

If V002 = 'No' or 'Don't know yet'

V003

You answered 'No' or 'Don't know yet' to the question about whether you would like to use a smartwatch to recognize cardiac arrest. What are the reasons for this?

Multiple answers are possible

Display answer options randomly

- I am healthy and therefore do not need it

- I am not afraid of cardiac arrest and therefore do not need it

- I don't want to be resuscitated in case of cardiac arrest

- I prefer not to think about the fact that I could have a cardiac arrest, and the smartwatch would always confront me with this

- It will probably be too expensive to have me monitored remotely for the onset of cardiac arrest

- I am afraid that my data will not be handled carefully

- I find it too complicated to use a smartwatch and/or smartphone

- I find it too complicated to sign up for this

- I have religious or cultural objections

- I am afraid that the technology will not work well enough and I will not be helped if I have a cardiac arrest

- I am afraid that the technology will not work well enough and then the ambulance or other emergency responders will show up at my door without anything happening

- I am afraid that the battery of my smartwatch will drain too quickly because of that feature

- Other, namely: <open answer field>

If V003 = more than one answer option

V004

Which of the following reasons is most important to you in your choice?

One answer option possible

- Load answer options Q003

- Load 'Other, namely:' option

- Don't know/no preference

V005

What would you be willing to spend per month maximum to be remotely monitored to recognize a cardiac arrest and summon emergency services?

Please enter an amount in euros

<open answer field> euro

**Part 4: Statements about smartwatch use**

T001

In the next block, you will see a number of statements about using a smartwatch to recognize cardiac arrest. For each statement, please indicate the extent to which you agree.

V006

I would like it if I could use a smartwatch to protect/monitor myself by recognizing a possible cardiac arrest.

| Totally disagree | Disadree | Neutral | Agree | Totally agree |
| --- | --- | --- | --- | --- |
|  |  |  |  |  |

V007

I would recommend this technology to family members and/or friends.

| Totally disagree | Disadree | Neutral | Agree | Totally agree |
| --- | --- | --- | --- | --- |
|  |  |  |  |  |

V008

The design (e.g., color and shape) of the smartwatch is important to me.

| Totally disagree | Disadree | Neutral | Agree | Totally agree |
| --- | --- | --- | --- | --- |
|  |  |  |  |  |

V009

The smartwatch should be waterproof so that I am also protected in the pool or shower.

| Totally disagree | Disadree | Neutral | Agree | Totally agree |
| --- | --- | --- | --- | --- |
|  |  |  |  |  |

V010

The technology (smartwatch and smartphone app) should be easy to use.

| Totally disagree | Disadree | Neutral | Agree | Totally agree |
| --- | --- | --- | --- | --- |
|  |  |  |  |  |

V011

I must be able to easily sign up and register for the ability to be monitored remotely.

| Totally disagree | Disadree | Neutral | Agree | Totally agree |
| --- | --- | --- | --- | --- |
|  |  |  |  |  |

V012

The technology to recognize cardiac arrest must work reliably.

| Totally disagree | Disadree | Neutral | Agree | Totally agree |
| --- | --- | --- | --- | --- |
|  |  |  |  |  |

V013

My data must be well protected.

| Totally disagree | Disadree | Neutral | Agree | Totally agree |
| --- | --- | --- | --- | --- |
|  |  |  |  |  |

V014

Through which channels would you like to be informed about the use of the smartwatch when this product is fully developed?

Multiple answers are possible

Display answers randomly

- My general practitioner / medical specialist

- Print advertisements

- Online advertisements

- Social media

- Digital newsletter

- Personal contact with the researchers

- TV

- Radio

- Other, namely: <open answer option>

- I would not like to be informed.

V015

Do you have any additional tips or comments you would like to give us for the development of the target technology?

<open response field>

**Part 5: Background questions**

A001

Which smartwatch are you currently using?

Display answer options randomly

- Apple

- Garmin

- Fitbit

- LG

- Casio

- Samsung

- Huawei

- Fossil

- Michael Kors

- Other, namely: <open answer field>

A002

Do you own a smartphone?

- Yes

- No

A003

What the highest level of education you have completed or the highest degree you have obtained?

- Lower than high school

- High school or similar

- Higher vocational education (HBO) or university but no degree

- HBO - bachelor

- HBO - master

- University - bachelor

- University - master

- University - PhD

- Other, namely: <Open answer field>

A004

Which of the following categories best describes your employment situation?

- Job, employed 1-34 hours per week on average

- Job, employed 35 hours or more per week on average

- No job, looking for work

- No job, not looking for work

- No job, cannot work due to illness or disability

- Retired

- Other, namely: <Open response field>

A005

What is your total gross household income per month?

Your answer will be processed anonymously

- Minimum (less than €1,600 per month)

- Below modal (€ 1,600 to € 2,600 per month)

- Modal (€ 2,600 to € 3,000 per month)

- One and a half times modal (€ 3,000 to € 4,000 per month)

- Twice modal (€ 4,000 to € 8,000 per month)

- More than 2x modal (8,000 per month or more)

- I don't know/won't say

A006

What is your background? Do you have a Dutch or a migration background?

- Dutch background

- Western migration background

- Non-Western migration background

- Other, namely: <open answer field>

- I do not want to say

A007

What is your marital status?

- Single

- In a relationship

- Married / registered partnership

- Widowed without a new relationship

- Divorced without a new relationship

- Other, namely: <open answer field>

A008

What is your household situation?

- Living with parents/family/guardian

- Single without children living at home

- Single with children living at home

- Living together and/or married without children living at home

- Living together and/or married with children living at home

- Other, namely: <open answer field>

# B. Translated Survey Patients

**Part 1: Introduction**

Dear Sir/Madam,

For research on a new technology to use smartwatches to recognize cardiac arrest, we are looking for patients who would like to give their opinions about it. The text in the next paragraph gives a little more background information.

In the Netherlands, 17,000 people are struck by cardiac arrest outside the hospital each year. A large number of these people die because the emergency services are called too late or not at all (if there are no witnesses). With every minute delay until proper treatment is started, the chance of making a good recovery drops significantly.

Researchers at the Amsterdam UMC are currently working on developing a new technology in which a smartwatch can recognize a cardiac arrest and immediately automatically summon the emergency services (ambulance service and citizen assistance).

The goal is that the technology will work on many different smartwatches and there is no need to buy a particular model. Anyone who owns a smartwatch and smartphone can then register and install the technology.

Completing this survey will take no more than 10 minutes of your time. Participation in the survey is completely voluntary and anonymous. By completing the survey, you declare your consent to the use of the information for research. The data will be processed confidentially. If you do not want to fill in an answer to a certain question, you do not have to.

Thank you very much for your cooperation!

**Inclusion criteria**

Are you part of the Care Panel of the Patient Federation?

- Yes

- No

Screenout if No

**Part 2: Screening**

In the next section, we ask some general questions. In addition, there are a number of questions related to your health and risk factors for developing cardiac arrest.

S001

What is your age?

<Open answer field>

S002

What is your gender? [Multiple-choice question, one answer possible]

- Male

- Female

- Other than the above

S003

What is your height?

<Open answer field, numbers only> in meters

S004

What is your weight?

<Open answer field, numbers only> in kg

Calculation BMI: (patients do not see this)

S005

Do you smoke? [Multiple-choice question, one answer possible]

- Yes

- No

- I have stopped

S006

Do you drink alcohol? [Multiple-choice question, one answer possible]

- Yes, on average less than 2 glasses of alcohol per week

- Yes, on average 2-7 glasses per week

- Yes, on average 7-14 glasses per week

- Yes, on average more than 14 glasses per week

- No, I never drank alcohol

- No, I have stopped

- Do not want to say

S007 [Multiple-choice question, one answer possible].

Do you have high cholesterol or are you taking medication for elevated cholesterol (e.g. simvastatin, atorvastatin, rosuvastatin, ezitimib, etc.)

- Yes

- No

- Do not know

S008

Do you have or had one or more of the following conditions? [Multiple-choice question, multiple answers possible]

- High blood pressure or are you taking blood pressure lowering medication

- Diabetes (diabetes)

- Cardiovascular disease (e.g., heart failure, myocardial infarction, heart valve defects, heart muscle disease, arrhythmia, congenital heart defects, calcification or narrowing of arteries, dilatation or bulging of a blood vessel, thrombosis or pulmonary embolism, etc.)

- Kidney damage

- Stroke (cerebral infarction, cerebral hemorrhage)

- Lung disease (e.g., asthma, chronic bronchitis, pulmonary emphysema, etc.)

- Other serious illness; namely <Open response field>

S009

Have close relatives (father, mother, brother or sister) experienced cardiovascular disease before age 55 (men) or 65 (women)?

- Yes

- No

- Do not know

S010

Have you ever experienced cardiac arrest yourself? [Multiple-choice question, one answer possible]

- Yes

- No

S011

Has anyone close to you ever had a cardiac arrest? [Multiple-choice question, one answer possible]

- Yes

- No

S012

If "yes" to the previous question,

Has anyone in your immediate family (father, mother, brother, sister, son or daughter) died of acute cardiac death/sudden cardiac death (someone who suddenly dropped dead with no known cardiovascular disease) [Multiple-choice question, one answer possible].

- Yes

- No

- Don't know

S013

Did you see with your own eyes that someone was in cardiac arrest? [Multiple-choice question, one answer possible]

- Yes

- No

S014

If "yes" to the previous question, Have you ever resuscitated someone? [Multiple-choice question, one answer possible]

- Yes

- No

S015

How did your experience of cardiac arrest/resuscitation affect you?

**Part 3: Perception**

In the next block, you will read some statements about using a smartwatch to recognize cardiac arrest. For each statement, please indicate the extent to which you agree.

V001a

What do you think about the idea of smartwatches being used to recognize cardiac arrest?

| Not interesting at all | Not interesting | Neutral | Interesting | Very interresting |
| --- | --- | --- | --- | --- |
|  |  |  |  |  |

V001b

Here you can explain your answer to the previous question.

<Open answer field>

V002

If it is possible in the future to reliably recognize cardiac arrest through a smartwatch, would you want to take advantage of this? [Multiple-choice question, one answer possible]

- Yes

- No

- Do not know yet

V003

<If "yes to the previous question>

Why would you want to use technology?

<Open answer field>

V004

If you answered "no" or don't know yet to the previous question, what are the reasons for that (multiple choices possible)? [Multiple-choice question, multiple answers possible]

- I am healthy

- I am not afraid of cardiac arrest

- I do not want to be resuscitated in case of cardiac arrest

- I do not want to think that I could have a cardiac arrest and this technology would always confront me with this

- I don't have a smartwatch and won't/can't buy one

- I don't have a smartwatch and don't want/can't buy a smartwatch

- It will be expensive to use this technology

- I am afraid that my data will not be handled carefully

- I find it too complicated to use a smartwatch and/or smartphone

- I have religious or cultural objections

- I am afraid it will not work properly and I will not be helped if I have a cardiac arrest

- I'm afraid it won't work properly and then emergency responders will show up at my doorstep without anything going on

- Other, namely: <Open response field>

V005

If you indicated multiple reasons in question the previous question, which one is most important to you in your choice?

- <Open answer field>/ answers from previous question

- Don't know/no preference

V006

What would you be willing to spend per month maximum to be remotely monitored to recognize a cardiac arrest and call the emergency services? [Amount in Euros]

<Open answer field>

V007

<If patient entered yes to question S009, S010 or S012 then>

Do your experiences with cardiac arrest influence your decision whether or not to use this technology in the future?

- Yes

- No

- Do not know

V008

<If patient "yes" to previous question> In what ways does it influence you?

<Open answer field>

**Part 4: Statements about smartwatch use**

In the next block, you will see a number of statements about using a smartwatch to recognize cardiac arrest. For each statement, please indicate the extent to which you agree.

V009

I would like to be able to use a smartwatch that recognizes a possible cardiac arrest of mine. [Likert scale]

| Totally disagree | Disadree | Neutral | Agree | Totally agree |
| --- | --- | --- | --- | --- |
|  |  |  |  |  |

V010

I would recommend this technology to family members and/or friends. [Likert scale]

| Totally disagree | Disadree | Neutral | Agree | Totally agree |
| --- | --- | --- | --- | --- |
|  |  |  |  |  |

V011

The technology (smartwatch and smartphone app) should be easy to use. [Likert scale]

| Totally disagree | Disadree | Neutral | Agree | Totally agree |
| --- | --- | --- | --- | --- |
|  |  |  |  |  |

V012

I need to be able to easily apply and register for the ability to be remotely monitored. [Likert scale]

| Totally disagree | Disadree | Neutral | Agree | Totally agree |
| --- | --- | --- | --- | --- |
|  |  |  |  |  |

V013

The technology to recognize cardiac arrest must work reliably.

| Totally disagree | Disadree | Neutral | Agree | Totally agree |
| --- | --- | --- | --- | --- |
|  |  |  |  |  |

V014

My data used with this technology/app should be well protected. [Likert scale]

| Totally disagree | Disadree | Neutral | Agree | Totally agree |
| --- | --- | --- | --- | --- |
|  |  |  |  |  |

V015

Which of the above statements do you think is most important?[Multiple-choice question, one answer possible].

- . The technology (smartwatch and smartphone app) should be easy to use.

- I should be able to easily sign up and enroll in the ability to be monitored remotely.

- The technology to recognize cardiac arrest must work reliably.

- My data must be well protected.

V016

Through which channels would you like to be informed about the use of the smartwatch when this product is fully developed? [Multiple-choice question, multiple answers possible]

- Via my general practitioner / medical specialist

- Via direct approach by the researchers via email or telephone

- Through advertisements

- Through social media

- Other, namely: <open answer option>

- I would not like to be informed.

- I do not know

V017

Do you have any additional tips or comments you would like to give us for the development of the target technology?

<open answer field>

**Part 5: Background questions**

A001

Do you own a smartwatch? [Multiple-choice question, one answer possible]

- Yes

- No

A002

If "Yes" to the previous question. Which smartwatch are you currently using? [Multiple-choice question, multiple answers possible]

- Apple

- Garmin

- Fitbit

- Samsung

- Huawei

- Fossil

- Michael Kors

- LG

- Casio

- Other, namely: <open answer field>

A003

Do you own a smartphone? [Multiple-choice question, one answer possible]

- Yes

- No

A004

What is the highest level of education you have completed or the highest degree you have obtained? [Multiple-choice question, one answer possible]

- Lower than high school

- High school or similar

- MBO

- Higher vocational education (HBO) or university but no degree

- HBO - bachelor

- HBO - master

- University - bachelor

- University - master

- University - PhD

- Other, namely: <Open answer field>

A005

Which of the following categories best describes your employment situation? ? [Multiple-choice question, one answer possible]

- Job, employed 1-34 hours per week on average

- Job, employed 35 hours or more per week on average

- No job, looking for work

- No job, not looking for work

- No job, cannot work due to illness or disability

- Retired

- Other, namely: <Open response field>

A006

What is your total gross household income per month? [Multiple-choice question, one answer possible]

- Minimum (less than €1,600 per month)

- Below modal (€1,600 to €2,600 per month)

- Modal (€ 2,600 to € 3,000 per month)

- One and a half times modal (€ 3,000 to € 4,000 per month)

- Twice modal (€ 4,000 to € 8,000 per month)

- More than 2x modal (8,000 per month or more)

- I don't know/won't say

A007

What is your background? Do you have a Dutch or a migration background? [Multiple-choice question, one answer possible]

- Dutch background

- Western migration background

- Non-Western migration background

- Other background

- I do not want to say

A008

What is your marital status? [Multiple-choice question, one answer possible]

- Single

- In a relationship

- Married / registered partnership

- Widowed without a new relationship

- Divorced without a new relationship

- Other, namely: <Open answer option>

A009

What is your living situation like? [Multiple-choice question, one answer possible]

- I live alone

- I live with my partner and/or children

- Other namely: <Open answer option>

A010

In which province do you live? [Multiple choice question, multiple answers possible]

- Drenthe

- Flevoland

- Friesland

- Gelderland

- Groningen

- Limburg

- North Brabant

- North Holland

- Overijssel

- Utrecht

- Zeeland

- South Holland

- Other, namely: <Open answer field>
